# Induction of innate immune responses by flagellin from the intracellular bacterium, ‘*Candidatus* Liberibacter solanacearum’

**DOI:** 10.1186/s12870-014-0211-9

**Published:** 2014-08-05

**Authors:** Guixia Hao, Marco Pitino, Fang Ding, Hong Lin, Ed Stover, Yongping Duan

**Affiliations:** 1 U. S. Horticultural Research Laboratory, USDA-ARS, 2001 South Rock Rd, Fort Pierce 34945, FL, USA; 2 San Joaquin Valley Agricultural Sciences Center, USDA-ARS, Parlier 93648, CA, USA

**Keywords:** Candidatus Liberibacter solanacearum, Flagellin, Flg22, Cell death, Reactive oxygen species, Gene expression

## Abstract

**Background:**

‘*Candidatus* Liberibacter solanacearum’ (Lso) is a phloem-limited alphaproteobacterium associated with the devastating zebra chip disease of potato (*Solanum tuberosum*). Like other members of Liberibacter, Lso-ZC1 encodes a flagellin domain-containing protein (Fla_*Lso*_) with a conserved 22 amino-acid peptide (flg22_*Lso*_). To understand the innate immune responses triggered by this unculturable intracellular bacterium, we studied the pathogen-associated molecular patterns (PAMPs) that triggered immunity in *Nicotiana benthamiana*, using the flg22_*Lso*_ peptide and the full length *fla*_*Lso*_ gene.

**Results:**

Our results showed that the expression of *fla*_*Lso*_ via *Agrobacterium*-mediated transient expression induced a slow necrotic cell death in the inoculated leaves of *N. benthamiana*, which was coupled with a burst of reactive oxygen species (ROS) production. Moreover, the expression of several representative genes involved in innate immunity was transiently up-regulated by the flg22_*Lso*_ in *N. benthamiana.* The Fla_*Lso*_, however, induced stronger up-regulation of these representative genes compared to the flg22_*Lso*_, especially that of flagellin receptor FLAGELLIN SENSING2 (*FLS2*) and respiratory burst oxidase (*RbohB*) in *N. benthamiana.* Although neither cell death nor ROS were induced by the synthetic flg22_*Lso*_, a weak callose deposition was observed in infiltrated leaves of tobacco, tomato, and potato plants.

**Conclusion:**

The flagellin of Lso and its functional domain, flg22_*Lso*_ share characteristics of pathogen-associated molecular patterns, and trigger unique innate immune responses in *N. benthamiana*. Slow and weak activation of the innate immune response in host plants by the flagellin of Lso may reflect the nature of its intracellular life cycle. Our findings provide new insights into the role of the Lso flagellin in the development of potato zebra chip disease and potential application in breeding for resistance.

## Background

Zebra chip (ZC) is an important potato disease causing millions of dollars in losses to both potato producers and processors in the United States [1]. The disease was first discovered in potato fields near Saltillo, Mexico in 1994, and was reported in Texas, USA in 2000 [2]. Since 2000, the disease has spread to several other states and has been accompanied by serious economic impacts [1]. The disease symptoms are characterized by necrotic flecking and medullary ray discolorations in tubers, leaf chlorosis, twisted stems, swollen nodes, vascular discoloration, leaf scorching, and wilting [3],[4]. The putative causal agent of Zebra chip disease is ‘*Candidatus* Liberibacter solanacearum’ (Lso) (also known as *Ca*. Liberibacter psyllaurous), which is transmitted by the potato psyllid, *Bactericera cockerelli*[3],[5]. Lso has a significantly reduced genome and shares a high degree of similarity with another important plant pathogen, ‘*Ca.* Liberibacter asiaticus’ (Las) [6],[7]. Lso is also associated with diseases of other crops including tomato, carrot (*Daucus carota L.*) in Finland, and celery (*Apium graveolens*) in Spain [3]. Currently all commercial potato cultivars appear to be susceptible to Lso infection and the only strategy for controlling the spread of the disease is by managing the potato psyllid [1].

Plant immunity relies on two levels of defense response against pathogens: pathogen-associated molecular patterns (PAMP)-triggered immunity (PTI) that is a component of basal defense and effector-triggered immunity (ETI) that reflects immunity to specific strains [8],[9]. PTI is associated with PAMP recognition and activation of plant plasma membrane receptors [8],[10]. Typically, PTI induces several important signaling pathways including calcium ion (Ca^2+^) influx, reactive oxygen species (ROS) production, and mitogen-activated protein kinase (MAPK) pathway activation [10]. Interactions between the peptide flg22, which is located at the N-terminal domain of a flagellin, and host surface-localized pattern recognition receptors flagellin sensing 2 (FLS2) in *Arabidopsis thaliana,* are well studied. In *A. thaliana,* FLS2 interacts with the receptor kinase BAK1 by forming a functional FLS2/BAK1 complex, which is subsequently followed by a typical PTI response [8],[10]. Orthologs of the *Arabidopsis* FLS2 have been identified in many plant species including *N. benthamiana*[11], *Lycopersicum esculentum*[12] and *Oryza japonica*[13]. Recently, it has been shown that silencing the expression of *NbFLS2* compromised the expression of downstream genes induced by flg22 [14]. In *Arabidopsis,* the ROS burst induced by the flg22 is regulated by *RbohD*[15]. In *N. benthamiana*, *RbohB*, a homolog of *RbohD,* is essential for ROS production and silencing of *NbRbohB* completely abolishes the ROS burst [14],[16]. In *N. benthamiana,* two mitogen-activated protein kinases (MAPKs), salicylic acid-induced protein kinase (SIPK), and wound-induced protein kinase (WIPK) are activated quickly after elicitation [17]. Furthermore, both SIPK and WIPK are essential for bacterial immunity in *N. benthamiana*[14].

It has recently been shown that another member of the Liberibacter genus, *Ca*. Liberibacter asiaticus (Las), encodes a functional flagellin that partially restored the motility of *Sinorhizobium meliloti fla* mutant and shares characteristics of pathogen-associated molecular patterns [18]. Like Las, the Lso genome contains the same number of open reading frames (30 ORFs) that are essential for the structure and assembly of a flagellum [6]. In this study, due to the inability to grow Lso in culture, we investigated the PAMP activity of Lso flagellin (Fla_*Lso*_) and its peptide flg22_*Lso*_*in planta* via infiltration or *Agrobacterium-mediated* transient expression. Our results demonstrate that transient expression of *fla*_*Lso*_ induces a delayed increase in ROS production and slow necrotic cell death in tobacco plants. Although the flg22 from *P. aeruginosa* (which was used as a control) induced the typical ROS bursts, the flg22_*Lso*_ peptide did not induce ROS production in tobacco, tomato, or potato, but did induce callose deposition in these three species. We further demonstrated that the peptide flg22_*Lso*_ and the flagellin, Fla_*Lso*_ induced expression of genes associated with PTI in *N. benthamiana*. These results provide new insights into the role of bacterial flagellin in the development of potato zebra chip disease.

## Results

### Lso encoding a flagellin with a conserved flg22_*Lso*_ peptide

**Figure 1:**
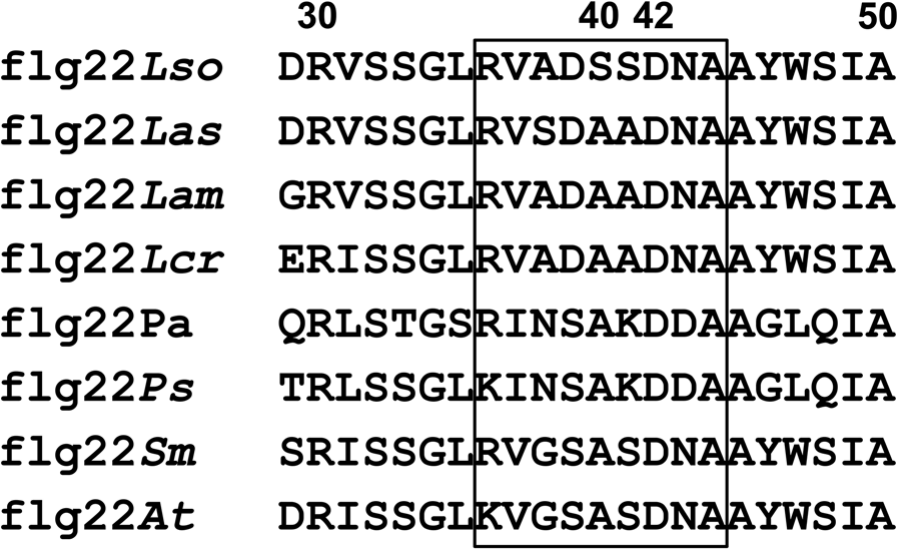
**Comparison of flg22 peptide sequence of ‘*Candidatus*Liberibacter solanacearum’ (GenBank accession number: YP_004062766.1) to the conserved flg22 from other bacteria.** Las: ‘*Ca.* Liberibacter asiaticus’ (GenBank accession number: YP_003064944.1), Lam: *Ca.* Liberibacter americanus (GenBank accession number: YP_00008798658.1), Lcr: *Liberibacter crescens* BT1 (GenBank accession number: YP_007233200.1), Pa: *Pseudomonas aeruginosa* (GenBank accession number: ADZ56326.1), Ps: *P. syringae* (GenBank accession number: WP_005738283.1), Sm: *Sinorhizobium meliloti* Rm1021 (GenBank accession number: NP_384775.1), At: *Agrobacterium tumefaciens* C58 (GenBank accession number: NP_353572.1). The central domain is shown in box.

In the Lso-ZC1 genome, several clusters of flagellar biosynthesis related genes were identified by sequence analysis. CKC_02645 was characterized as encoding a flagellin domain-containing protein. This gene contains 1374 nucleotides and encodes a 457 amino-acid protein, designated as Fla_*Lso*_. Fla_*Lso*_ shares 61% identity to the flagellin from *Ca*. Liberibacter asiaticus (Las), 59% identity to the flagellin from *Ca.* Liberibacter americanus (Lam) and 51% identity to the flagellin from *L. crescens* BT1. A conserved flagellin domain was identified consisting of 22 amino acids located at position 29 to 50 at the N terminus of Fla_*Lso*_ and was designated as flg22_*Lso*_. Flg22_*Lso*_ shares high identity (86%) to the flg22 peptides from Las, Lam and L. crescens BT1and it shares 77% identity to the flg22 from the closely related species *Agrobacterium tumefaciens* and *Sinorhizobium meliloti* 1021, which do not induce plant immune responses [19],[20]. Flg22_*Lso*_ shares 41% identity with the flg22 from *Pseudomonas aeruginosa* and 55% identity with the flg22 from *Pseudomonas syringae pv. tabaci*, which trigger a strong nonhost innate immune response [21]. In flg22, the amino acid residue D42 is critical for its PAMP activity [22],[23]. Although flg22_*Lso*_ possesses amino acid D42, only two other amino acids are conserved in the central RINSAKDDA motif (Figure 1).

### Slow necrotic cell death induced by transient expression of the *fla*_*Lso*_ in *N. benthamiana*

**Figure 2:**
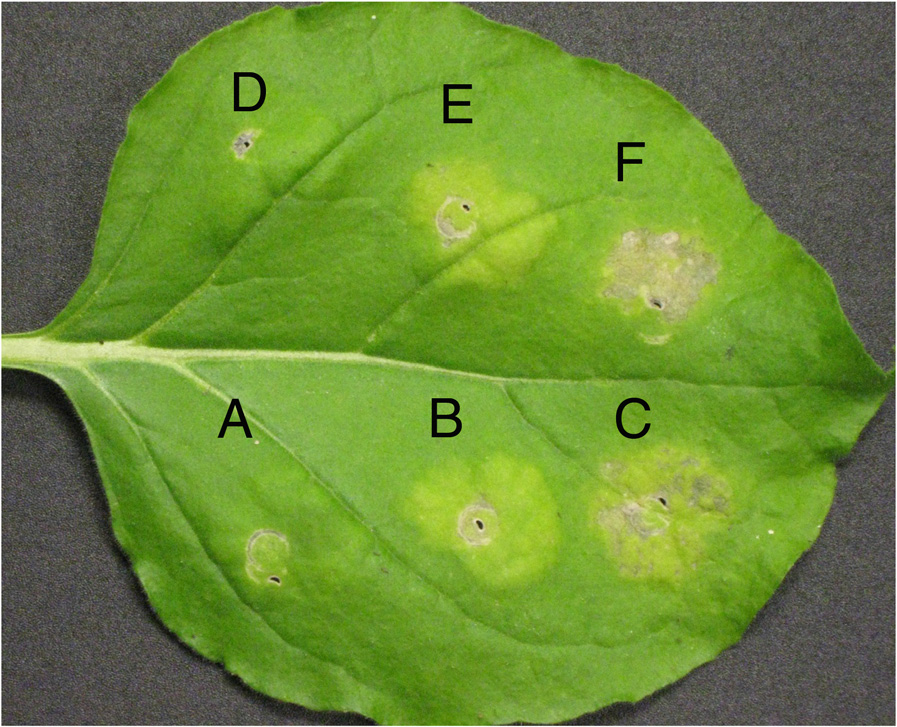
**Cell death in*N. benthamiana*leaves induced by transient expression of the*fla***_***Lso***_**,‘*Ca.*Liberibacter solanacearum’ flagellin gene.** Transient expression of *Agrobacterium tumefaciens* strain GV3101 alone, GV3101 containing the pBin vector, or GV3101 containing pBin:*fla*_*Lso*_ at an OD_600_ ~ 0.8 **(A, B, C)** and 1.2 **(D, E, F)**, respectively. Photographs were taken ten days after infiltration. The experiments were performed in six independent replicates.

For the transient expression assay of *fla*_*Lso*_ in *N. benthamiana*, various concentrations of *A. tumefaciens* GV3101 with and without *fla*_*Lso*_ were tested. At an OD_600_ of 0.8 and 1.2, necrotic cell death was observed in the infiltration zone with pBin:*fla*_*Lso*_ at 8 days after inoculation; the control with pBin vector alone exhibited chlorosis but not necrosis, and GV3101 with no vector showed no visible response (Figure 2). In addition, cell death was measured by electrolyte leakage from leaf discs infiltrated with MgCl_2_, GV3101 carrying pBin vector and pBin:*fla*_*Lso*_ constructs. Overexpression of the *fla*_*Lso*_ in tobacco leaves increased electrolyte leakage on 4 and 5 dpi (Additional file 1: Figure S1). Taken together, this indicates that the expression of the flagellin gene, *fla*_*Lso*_, causes necrotic cell death in the infiltrated zone of *N. benthamiana*. However, in tomato and potato inoculations, no obvious differences were observed after inoculation with various concentration of GV3101 containing the pBin vector alone or pBin:*fla*_*Lso*_, respectively (data not shown).

### Callose deposition induced by flg22_*Lso*_ and flg22_*Las*_

**Figure 3:**
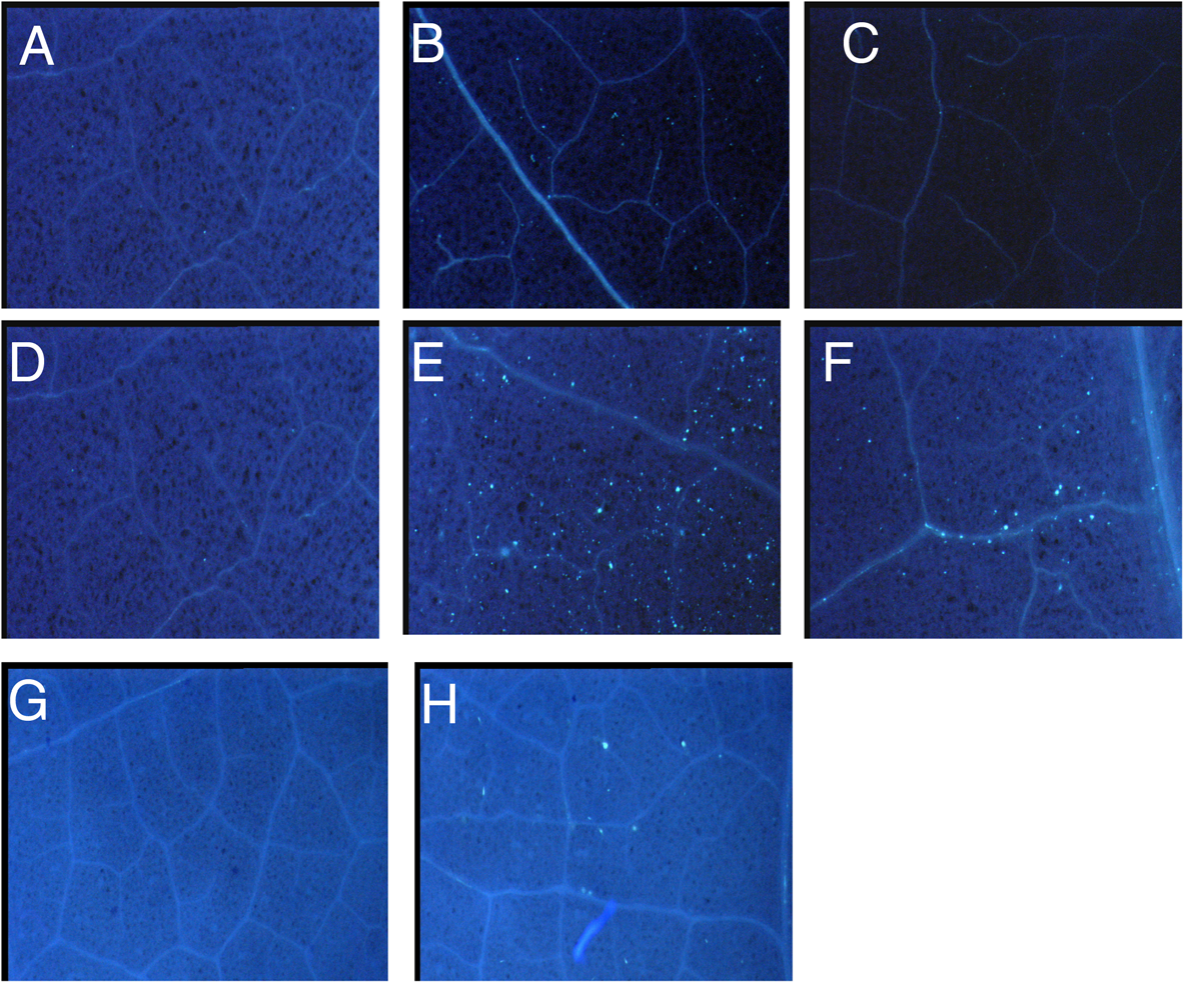
**Microscopic detection of callose deposition.***N. benthamiana***(A, B and C)** and tomato **(D, E and F)** infiltrated with the water control, flg22_*Lso*_ and flg22_*Las*_, respectively. Potato **(G and H)** infiltrated with water and flg22_*Lso*_, respectively. Leaves were collected at 24 h post infiltration and then stained with aniline blue. Callose deposition was observed under UV epifluorescence microscope.

The conserved flagellin domain flg22_*Las*_ from *Ca.* Liberibacter asiaticus induced callose deposition when infiltrated into tobacco [18]. Compared to flg22_*Las*_, flg22_*Lso*_ has three amino acid substitutions including a serine (S) to alanine (A) at position 38, and alanine (A) to serine (S) substitutions at positions 40 and 41 (Figure 1). Both synthetic peptides flg22_*Lso*_ and flg22_*Las*_ at a concentration of 40 μM were infiltrated into leaves of *N. benthamiana*, tomato and potato. Neither peptide induced cell death in the infiltrated zone but callose deposition was observed in infiltrated *N. benthamiana*, tomato and potato, with more callose deposition from flg22_*Lso*_ compared to flg22_*Las*_ in both tobacco and tomato (Figure 3). Compared to tobacco and potato, a more robust callose deposition was observed in tomato after treatment with either one of the peptides (Figure 3E and F).

### ROS production induced by the *fla*_*Lso*_, but not by the flg22_*Lso*_ in *N. benthamiana*

**Figure 4:**
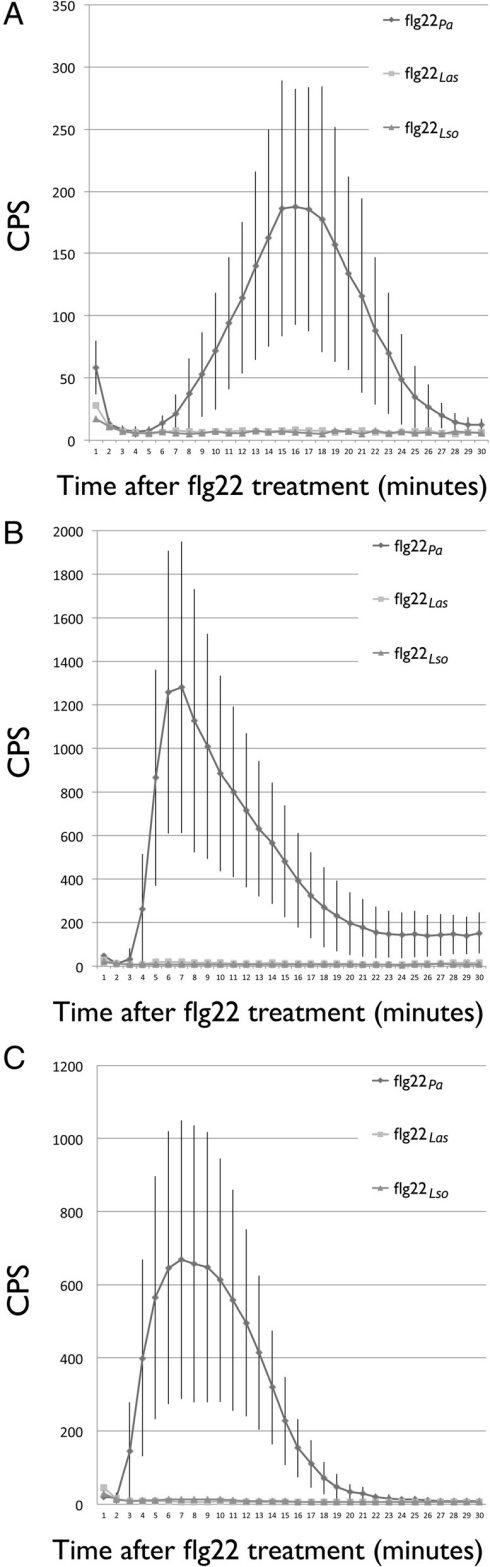
**ROS assay for flagellin peptide treated plants.***N. benthamiana***(A)**, potato **(B)** and tomato **(C)** treated with flg22 from *P. aeruginosa* showed typical ROS response. No ROS response was seen with flg22_*Las*_ or flg22_*Lso*_ treated plants. CPS: count per second.

**Figure 5:**
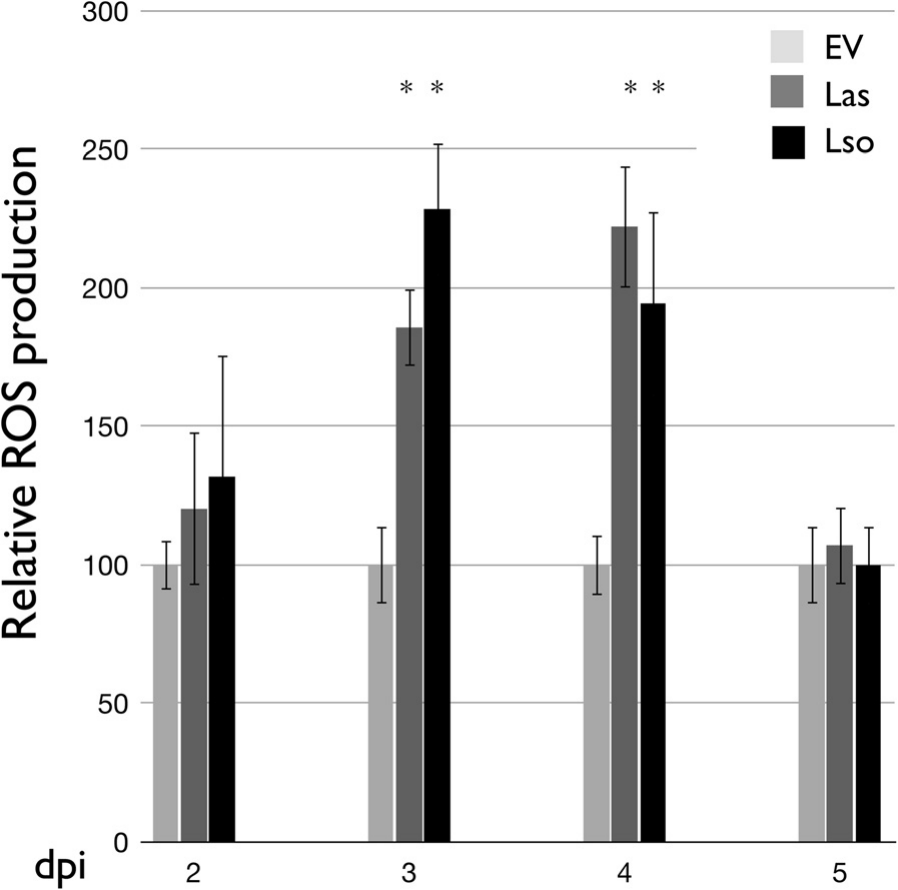
**ROS assay for the*fla***_***Lso***_**and the*fla***_***Las***_**infiltrated zones of*N. benthamiana*leaves.** Data shown are relative values (error bars represent standard error of the mean based on four samples from four different plants) at 2, 3, 4 and 5 dpi after infiltration with *Agrobacterium tumefaciens* strain GV3101 containing EV: Empty vector, pBin; Las: pBin:*fla*_*Las*_ and Lso: pBin:*fla*_*Lso*_. * marked as significant difference by student *t*-test.

One of the important events of PTI response is a rapid and transient burst of ROS production. We examined whether ROS production is induced by flg22_*Lso*_ or flg22_*Las*_. Neither flg22_*Lso*_ nor flg22_*Las*_ produced an ROS burst when infiltrated at 0.1 μM, 1 μM, 10 μM or 40 μM in tobacco, tomato or potato; while the control flg22 from *P. aeruginosa* shows a typical ROS production at a 0.1 μM concentration in the three plant species (Figure 4). In contrast, ROS production was detected in transient expression of *N. benthamiana* leaves infiltrated with *A. tumefasciens* containing pBin:*fla*_*Lso*_ and pBin:*fla*_*Las*_ clones respectively (Figure 5). The strongest ROS response was observed on the third and fourth day after infiltration. However, no ROS response was detected in tomato or potato infiltrated with these peptides and constructs (data not shown), which is consistent with the observation that no obvious cell death was induced in these plants.

### PTI gene expression transiently up-regulated by flg22_*Lso*_ in *N. benthamiana*

**Figure 6:**
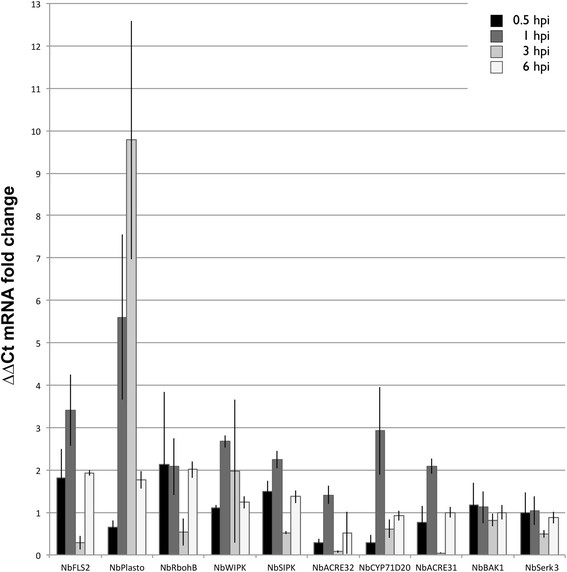
**Flg22**_***Lso***_**-triggered changes in the expression of genes related to plant defense in*N. benthamiana*.***N. benthamiana* leaves were infiltrated with flg22_*Lso*_ at 40 μM and water as a control. Leaf tissue was harvested from infiltrated spots at 0.5, 1, 3 and 6 h post-infiltration (hpi) for RNA isolation and cDNA preparation. RT-qPCR was performed to check gene expression of *NbFLS2, NbWIPK, NbSIPK, NbBAK1*, *NbRbohB*, *NbCYP71D20*, *NbACRE31*, *NbACRE32, NbPlastocyanin* and *NbSerk3*. The samples were normalized against *NbEF1*α. Data were shown as average fold gene induction in response to water infiltrated samples from three independent experiments.

The expression of many plant genes is up-regulated in PAMP-triggered immunity [24],[25]. To investigate expression levels of PAMP-associated genes in *N. benthamiana* after flg22_*Lso*_ infiltration, reverse transcriptase quantitative PCR (RT-qPCR) was performed. After *N. benthamiana* was infiltrated with the flg22_*Lso*_ peptide, the transcript abundance was measured at 0.5, 1.0, 3.0, and 6.0 hours post infiltration (hpi). The expression of *NbFLS2* was up-regulated approximately 2 fold at 0.5 hpi, more than 3 fold at 1.0 hpi, and then decreased at 3.0 hpi with flg22_*Lso*_ treatment (Figure 6). Previously, three marker genes in *N. benthamiana*, *NbCYP71D20*, *NbACRE31* and *NbACRE32*, were found to rapidly increase following flg22_*Psy*_ treatment [14],[26]. These three PAMP-marker genes were transiently induced by flg22_*Lso*_ at 1.0 hpi and then subsequently declined in our RT-qPCR assay, which indicates that flg22_*Lso*_ induces a typical transient PAMP triggered immunity gene response. FLS2 directly binds bacterial flagellin and then interacts with BAK1 to form a recognition complex [8]. However, the expression level of *NbBAK1* showed no obvious differences in our assay. Somatic embryogenesis receptor kinase 3 (*NbSerk3*)/BAK1 is required for PAMP-triggered immunity in *N. benthamiana*[26]. The expression pattern of *NbSerk3*/ *NbBAK1* did not show an obvious change upon flg22_*Lso*_ treatment. Two MAPKs, *NbWIPK* and *NbSIPK*, were transiently increased at 1.0 hpi in our experiment. *NbRbohB* expression was up-regulated about 2 fold except at 3.0 hpi. Notably, most of these PTI related genes are up-regulated at 0.5 hpi or 1.0 hpi, and then diminish at 3.0 hpi. Plastocyanin, which plays a key role in photosynthesis, was reported to be induced in the PTI response to nonpathogenic *P. fluorescens*[11]. In our experiment, transcript abundance of *NbPlastocyanin* increased 5 fold at 1.0 hpi and almost 10 fold at 3.0 hpi (Figure 6). Taken together, our results showed that flg22_*Lso*_ transiently induced PAMP-triggered gene expression in *N. benthamiana*.

### Long lasting PTI gene expression induced by *fla*_*Lso*_ in *N. benthamiana*

**Figure 7:**
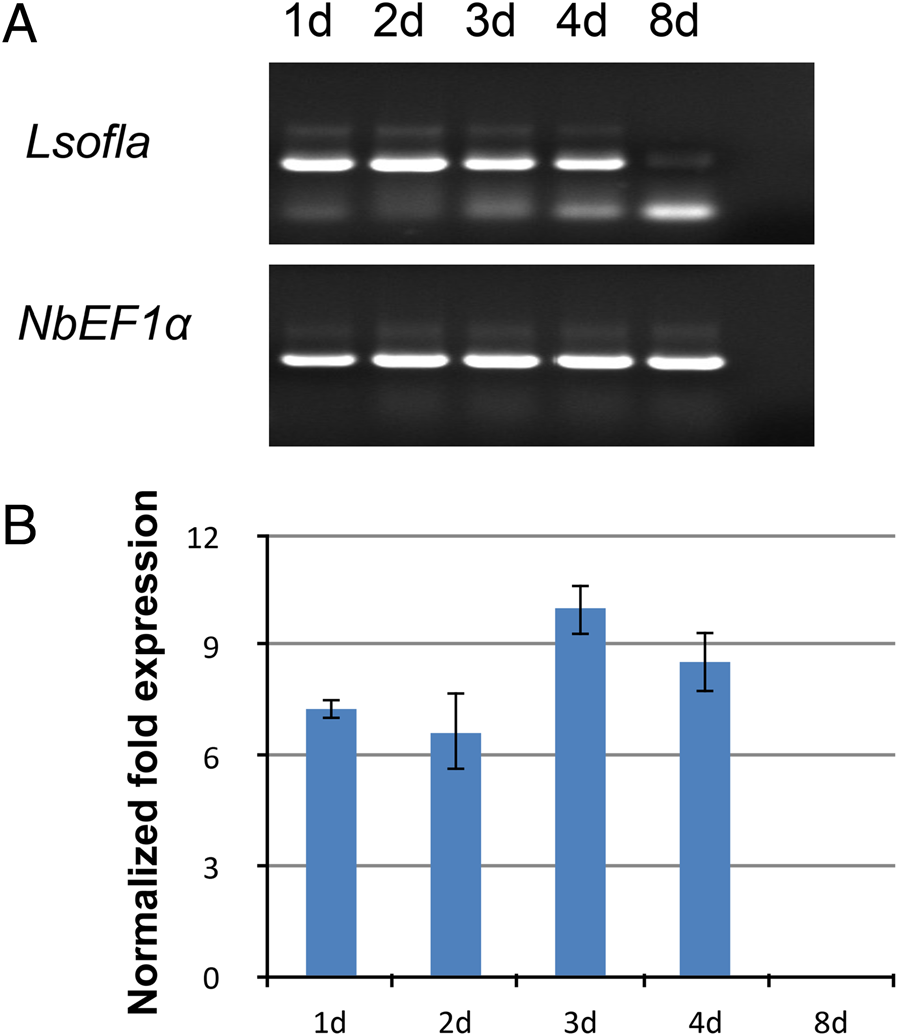
**Transient expression of the*fla***_***Lso***_**in*N. benthamiana*.***N. benthamiana* leaves were inoculated with *A. tumefaciens* GV3101 containing pBin:*fla*_*Lso*_. The infiltrated tissues were harvested at 1, 2, 3, 4 and 8 days post-infiltration (dpi) for RNA isolation and cDNA preparation. RT-PCR **(A)** and RT-qPCR **(B)** were performed to determine gene expression of the *fla*_*Lso*_ at different time after infiltration. For RT-qPCR, the samples were normalized against *NbEF1*α. Data were shown as average fold change from three independent experiments.

**Figure 8:**
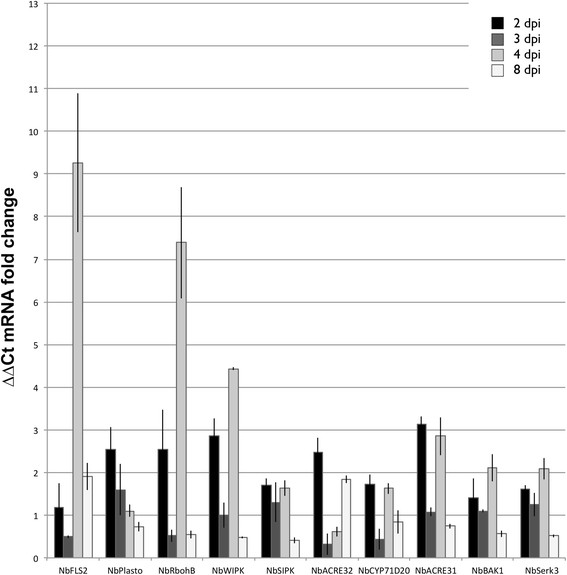
**Transient expression of the*fla***_***Lso***_**triggered changes in the expression of genes related to plant defense in*N. benthamiana*.***N. benthamiana* leaves were inoculated with *A. tumefaciens* GV3101 containing pBin:*fla*_*Lso*_ and GV3101 containing empty vector pBin as a control. The infiltrated spots were harvested at 2, 3, 4 and 8 days post-infiltration (dpi) for RNA isolation and cDNA preparation. RT-qPCR was performed to check gene expression of *NbFLS2, NbWIPK, NbSIPK, NbBAK1*, *NbRbohB*, *NbCYP71D20*, *NbACRE31*, *NbACRE32, NbPlastocyanin* and *NbSerk3*. The samples were normalized against *NbEF1*α. Data were shown as average fold gene induction in response to vector infiltrated samples from three independent experiments.

The expression of *fla*_*Lso*_ and its elicitation of PAMP-associated gene expression in *N. benthamiana* were investigated after infiltration with *A. tumefasciens* containing pBin:*fla*_*Lso*_ or the empty pBin vector plasmid. A high level of expression of *fla*_*Lso*_ was indicated by a strong band from 1 to 4 days after inoculation (dpi). A very faint band was observed on 8 dpi (Figure 7A). RT-qPCR data further showed that the transcript abundance after inoculation increased to its highest level on 3 dpi and decreased to an undetectable level compared to the host reference gene *NbEF1*α (Figure 7B). Meanwhile, the gene expression levels of PAMP-associated genes were measured at 2, 3, 4, and 8 dpi (Figure 8). RT-qPCR data showed that the expression of *NbFLS2* was up-regulated more than 9 fold at 4 dpi. The abundance of *NbBAK1*/*NbSerk3* was increased about 2 fold on 4 dpi. The expression of three other PAMP-associated genes, *NbCYP71D20*, *NbACRE31* and *NbACRE32* were upregulated and varied only slightly at different time after infiltration. Two MAPKs, especially *NbWIPK,* were up-regulated more than 4 fold at 4 dpi. *NbRbohB* dramatically increased at 4 dpi, which agreed with our ROS assay results that the strongest ROS production was detected at 4 dpi. The expression level of plastocyanin gradually declined over the evaluation period, in contrast to the transient high induction at 1.0 and 3.0 hpi observed with flg22_*Lso*_. Collectively, the expression of PAMP-associated genes elicited by the *fla*_*Lso*_ was different from the transient up-regulation with peptide flg22_*Lso*_ induction. Most of these genes were strongly induced at 4 dpi, and then decreased, which correlated with the expression pattern of the *fla*_*Lso*_ in *N. benthamiana*.

## Discussion

Our results revealed that transient expression of the Lso flagellin gene (*fla*_*Lso*_) induced a burst of ROS and slow necrotic cell death in *N. benthamiana* plants but not in the tomato or potato plants tested. Although the peptide flg22_*Lso*_ did not induce cell death or a ROS reaction, it did induce callose deposition in all three plant species*.* We determined that the expression of PAMP-triggered genes was transiently up-regulated with synthesized peptide flg22_*Lso*_ treatment. We further showed that these genes were more strongly affected by *Agrobacterium*-mediated transient expression of the full length gene, *fla*_*Lso*_. Our results demonstrate that Lso flagellin and its short peptide, flg22_*Lso*_ have a PAMP activity, and both of them trigger PTI response.

### Callose deposition induced by Lso and Las peptides

Many reports document that flg22_*Lso*_ peptides can induce plant defense responses, including reactive oxygen species (ROS), pathogenesis related gene expression and callose deposition [27]. Amino acid D42 of flg22_*Lso*_ has been demonstrated to be essential for the elicitor activity of *Xanthomonas* and *P. syringe* pv. *tabaci* in non-host species [22],[23]. In our study, flg22_*Lso*_*,* which contains D42, did not induce visible cell death or ROS production in the plants tested, however, it was found to induce callose deposition in tobacco, tomato and potato plants. This suggests that flg22_*Lso*_ may interact with the FLS2 receptors of all three plant species. In addition, we screened over one hundred potato genotypes using flg22_*Lso*_ and flg22_*Las*_. We found that flg22_*Lso*_ and flg22_*Las*_ induced ROS response in several potato cultivars and the full flagellin gene has the ability to interact with the potato FLS2 receptor in yeast two hybridization assay (Duan, unpublished data). The flg22 peptides from *A. tumefaciens* and *S. meliloti* posses the D42 amino acid but did not show PAMP activity [27]. This indicates that other amino acids are also important for flg22 elicitor activity in addition to D42. Amino acid changes made at S38 and A39 completely abolished the PAMP activity of flg22_*Las*_, indicating that serine and aspartate at these positions are essential for flg22_*Las*_ recognition by tobacco plants [18]. Compared to flg22_*Las*_, flg22_*Lso*_ has three amino acid substitutions and induces more callose deposition than flg22_*Las*_ in tobacco and tomato. It is worth noting that callose deposition was observed although no ROS production was detected in the infiltrated plants with either flg22_*Lso*_ or flg22_*Las*_. However, callose deposition is sometimes seen in a host plant’s response to pathogen infection as well as being a component of non-host resistance, and high levels of callose are formed in response to Las infection in citrus [28].

### Necrotic cell death induced by the *fla*_*Lso*_ in *N. benthamiana*

Flagellin is a bacterial elicitor associated with the induction of plant and animal defense responses. In our study, we discovered that transient expression of the *fla*_*Lso*_ induced necrotic cell death in tobacco, which was much slower than that reported for a typical hypersensitive response (HR) [29]. Transient expression of *P. syringae* flagellin FliC gene with or without signal peptide induced ROS and FLS2-dependent immunity in a non-host, but did not induce cell death within five days [30]. In our infiltration experiments, the expression of *fla*_*Lso*_ reached the highest level on 3 dpi and decreased to a very low level on 8 dpi, however necrotic cell death was not observed until 8–10 days after infiltration. This resembles cell death in a compatible pathogen-host interaction, which is much slower than non-host responses. Recently it has been reported that tobacco (*N. tabacum*) is a host for Lso [31]: response of a weak host rather than a non-host may explain our observation of necrotic cell death rather than a HR, which is characterized as rapid, localized plant cell death [29]. In contrast, perception of PAMPs leads to small metabolic changes during PTI so that energy production can be sustained in the presence of PAMPs [32]. It is intriguing that transient expression of the full length *fla*_*Lso*_ inside plant cells could also induce up-regulation of PAMP-associated gene expression, ROS response and slow necrotic cell death. FLS2 is a receptor that is known to recognize extracellular flagellin. In mammals, PAMPs such as flagellin in the cytosol induce a localized cell-death-associated immune response to pathogens. However, no cytoplasmic nucleotide-binding leucine-rich (NLR) receptor or other cytoplasmic receptor was identified in plants [31]. Most studies on plant FLS2 have focused on interaction with flg22 of flagellin administered extracellularly [27], as would occur with infection by *Pseudomonas* and many other bacterial pathogens. *Candidatus* Liberibacter species are strictly intracellular, and therefore, in order to function as a PAMP, their flagellins would require recognition within the plant cell. It has been demonstrated that transgenic expression of the flagellin from a rice-incompatible strain of *Acidovorax avenae* induced immune responses, expression of defense related genes, production of hydrogen peroxide and cell death in rice plants [33]. In another study, when FLS2-GFP was transgenically expressed, the FLS2 was primarily localized on the cell membrane before binding to flg22. After binding, the FLS2 accumulated into intracellular mobile vesicles and degraded upon flg22 activation [34]. In addition to being localized on cell membranes, FLS2 was also observed in intracellular vesicles of various sizes and shapes in a protoplast assay [35]. All of these together indicate that there is a unique pathway involving the interaction between FLS2 and Lso flagellin inside a plant cell. Further research is necessary to understand this pathway and downstream host response.

### PTI gene expression responses induced by Lso flagellin and peptide

As a complement to the observations discussed above, we investigated flg22_*Lso*_-induced gene expression following infiltration into *N. benthamiana. NbFLS2* and three other PAMP-marker genes, *NbCYP71D20*, *NbACRE31* and *NbACRE32*, were shown to be rapidly and transiently up-regulated (Figure 6) as was previously reported with the flg22 from *Pseudomonas*[14],[26]. We also assessed several other genes which are reported to be associated with different host-defense systems. ACRE (Avr9/Cf9 rapidly elicited) genes associated with race-specific defense responses were found to be up-regulated by the infiltration of flg22_*Lso*_ in *N. benthamiana*, similar to the result seen when resistant-race tobacco cells (Cf9 genotype) are treated with the fungal elicitor Avr9 [24].

When *P. fluorescens* was infiltrated into non-host *N. benthamiana*, the plastocyanin gene was markedly up-regulated, and silencing this gene compromised PTI [11]. Plastocyanin is a small Cu-containing protein which acts as an electron carrier between the cytochrome *b*_*6*_*f* and photosystem 1 complexes in the photosynthetic electron-transfer chain [11]. Surprisingly, our results showed that the expression of *NbPlastocyanin* was the most highly induced gene in the PTI response by flg22_*Lso*_ treatment. However, the up-regulation of *NbPlastocyanin* expression gradually decreased over several days with *Agrobacterium*-mediated expression of full length *fla*_*Lso*_, which may have resulted from impaired chloroplast function as cell death was slowly induced. It has been reported that there is cross-talk between PAMP-triggered immunity and photosynthesis [32]. Though a typical pattern of PAMP-triggered gene expression was observed with flg22_*Lso*_ infiltration, no ROS production was detected. This may be explained by the fact that two different signaling pathways were separated after calcium influx initiation: one leading to the ROS burst and the other to MAPKs and other gene activation [14]. It was reported that domains other than flg22 contribute very little to the elicitation of the FLS2-mediated *Arabidopsis* defense response [22]. However, a second region designated as flgII-28 within flagellin apart from flg22 was recently identified in *P. syringae pv. tomato* and flgII-28 was shown to induce ROS in *N. benthamiana* not in *Arabidopsis* or bean [36]. Furthermore, the allelic variation of flg22 and flgII-28 were reported to affect the plant immune response significantly, and to have no effect on bacterial motility [36]. This may explain our findings that the full flagellin gene, *fla*_*Lso*_, not the peptide flg22_*Lso*_, has the ability to induce ROS production and slow cell death in tobacco, which suggests other flagellin domains such as flgII-28 or post-translational modifications may also be associated with plant defense response. It is important to point out that transient expression of the *fla*_*Lso*_ only induced ROS production in tobacco but did not lead to ROS reaction in tested tomato and potato plants. This could be explained by the sequence variations within the FLS2 genes among species/varieties of host plants (Duan unpublished data).

It is established that FLS2 directly binds to flagellin in the *Pseudomonas* non-host system [27] and then interacts with BAK1 to form the FLS2/BAK1 complex, which activates the downstream signaling such as MAPK pathway. In our experiments, the expression of two MAPKs, *NbWIPK* and *NbSIPK* were indeed up-regulated in *N. benthamiana* by the *fla*_*Lso*_ (Figure 8). The prolonged activation of a MAPK pathway in cells may cause a redox imbalance and generate ROS, eventually leading to cell death [37]. Since MAP kinases are primarily regulated at protein levels, the levels of these proteins in *N. benthamiana* after inoculation with *flg*22_*Lso*_ and or *fla*_*Lso*_ need a further investigation.

PAMP-triggered immunity is important for plants to limit pathogen growth or generate signals for adaptation to secondary infections [38]. PAMPs, including flg22, activate components of the salicylic acid and jasmonic acid defense pathways, which protect against potential pathogenic bacteria [39]. The molecular events that occur during PTI and elicitor-triggered immunity (ETI) partially overlap including SA, ROS, and activation of MAPK cascades [40]. However, ETI elicits a much stronger response than PTI, indicating a quantitative difference between these two immunity responses [8]. The discovery that the Fla_*Lso*_ and flg22_*Lso*_ have PAMP activity and trigger PTI reveals that the flagellin from intracellular bacteria can initiate plant defense responses. The identification of a compatible FLS2 is critical for the development of potato plants with increased resistance against *Ca*. L. solanacearum via marker-assisted conventional breeding and genetic engineering.

## Conclusion

Zebra chip (ZC) is an important potato disease associated with the phloem-limited intracellular bacterium ‘*Candidatus* Liberibacter solanacearum’ (Lso). In this study, we examined the PAMP activity of the flagellin of Lso and its functional domain, flg22_*Lso*_*in planta*. We found that flg22_*Lso*_ has the ability to induce callose deposition and it also triggers transient up-regulation of PTI associated genes in *N. benthamiana*. We determined that the expression of *NbFLS2* and three marker genes, *NbCYP71D20*, *NbACRE31* and *NbACRE32,* are rapidly upregulated. Surprisingly we found the expression of *NbPlastocyanin* increased dramatically, rising by 10 fold at 3 hpi. However, neither cell death nor ROS were induced by the flg22_*Lso*_. We also determined that expression of the full length flagellin gene induces a much stronger PTI response compared to peptide flg22_*Lso*_, especially upregulation of the PAMP-associated genes *NbFLS2* and *NbRbohB*. In addition, the expression of *fla*_*Lso*_ induced ROS production and necrotic cell death in *N. benthamiana* via *Agrobacterium*-mediated transient expression. The discovery that the Fla_*Lso*_ and its short peptide have PAMP activity and the ability to trigger PTI provide new insights into the role of bacterial flagellin in the development of potato zebra chip disease and a potential application in breeding for resistance.

## Methods

### Plants and bacteria cultivation

Seeds of *N. benthamiana* and tomato were germinated in chambers with cycles of 16 h light and 8 h dark at 26°C. The seedlings were then transferred into Fafard® 4P Mix soil in plastic containers and grown in greenhouses. Potato cultivar (Atlantic) was planted directly in Fafard® 4P Mix soil in plastic containers and grown in greenhouses.

*E. coli* was grown at 37°C and *A. tumefaciens* was grown at 28°C in Luria-Bertani (LB) medium. Kanamycin (kan) was added to the medium at a concentration of 50 μg/mL.

### *fla*_*Lso*_ construction for transient expression in plants

The full length gene of *fla*_*Lso*_ was amplified using genomic DNA as a template with primers *fla*_*Lso*_F and *fla*_*Lso*_R (5′-AAA*CCCGGG*TGTCATTTCGATTTTTAAGGATA-3′; 5′- AAA*CCCGGG*CTAACCACGGAAAAGAGATAGAATT-3′). Italicized bases are SmaΙ restriction sites included for cloning. The PCR product was ligated into PCR2.1-TOPO vector and transformed chemically into *E. coli* TOPO10 cells following the manufacture’s instruction (Invitrogen, Carlsbad, CA). The positive clones were used for plasmid isolation and sequence verification. The consensus clone was digested, gel purified and cloned into a binary vector pBINPLUS/ARS-2x35S (pBin) generating pBin:*fla*_*Lso*_. The recombinant vector was then transformed into *A. tumefaciens* GV3101 by a freeze-thaw method [41].

### Peptide infiltration and callose deposition assay

The peptides of flg22_*Lso*_ (DRVSSGLRVADSSDNAAYWSIA) and flg22_*Las*_ (DRVSSGLRVSDAADNAAYWSIA) were synthesized by Life Tein Company (South Plainfield, NJ). Synthetic peptides were diluted with double distilled water to a final concentration of 40 μM and infiltrated into plant leaves with a 1 mL needleless syringe. At the second day post-infiltration (dpi), callose deposition was detected with aniline staining as described previously [39]. Briefly, the tissue was cleared and dehydrated in 100% ethanol. Cleared leaves were washed with distilled water and then stained overnight at room temperature in 0.01% aniline blue in 67 mM K_2_HPO_4_ (pH 12). Stained material was mounted in 50% glycerol and observed under ultraviolet of epifluorescence (Leitz DMR microscope, Leica Microsystems, Buffalo Grove, IL).

### Plant infiltration and RNA isolation

*A. tumefaciens* GV3101 and *A. tumefaciens* GV3101 containing vector pBin or pBin:*fla*_*Lso*_ were cultured over night in 2 mL of LB medium with the addition of 50 μg/mL kan. Fifty microliter of the overnight cultures were inoculated into fresh 5 mL LB medium for another 24 hr with the addition of 50 μg/mL kan, 10 mM MES (2-(*N*-morpholino)-ethanesulfonic acid), and 100 μM acetosyringone. The overnight cultures were centrifuged, washed, and re-suspended in Agromix (10 mM MgCl_2_, 10 mM MES, and 100 μM acetosyringone). The suspension was adjusted to different OD_600_ values with Agromix and kept at room temperature for at least 3 hr. The final cell suspension was used to inoculate plant leaves with a 1 ml needleless syringe.

*N. benthamiana* leaves were infiltrated with flg22_*Lso*_ at 40 μM and water as a control. As flg22_*Lso*_ induced rapid and transient PAMP-triggered gene expression [14], the infiltrated zones were harvested at 0.5, 1, 3 and 6 h post-infiltration (hpi) for RNA isolation. For full *fla*_*Lso*_ transient expression, *A. tumefaciens* GV3101 containing vector pBin or pBin:*fla*_*Lso*_ was used to infiltrate *N. benthamiana* leaves at OD_600_ ~ 0.5. Similarly the infiltrated zones were harvested at 2, 3, 4 and 8 d post-infiltration (dpi) for RNA isolation because cell death was observed after 8 to 10 dpi. Trizol reagent was used for RNA extraction according to the manufacture’s instructions (Sigma-Aldrich, St. Louis, MO). Total RNA was quantified using the Nanodrop and treated with RQ1 RNase-free DNase from Promega Corp (Madison, WI).

### ROS assay and ion-leakage assay

For the flg22_*Lso*_ and flg22_*Las*_ peptides, leaf discs of *N. benthamiana*, tomato and potato were floated on water overnight. Prior to ROS measurement, water was replaced with 100 μl of assay solution (17 mM lumino, 1 μM horseradish peroxidase, flg22_*Lso*_ or flg22_*Las*_ at concentrations of 0.1 μM, 1 μM, 10 μM and 40 μM). Luminescence was measured using the Perkin Elmer Victor3 V 1420 Multilabel Plate Counter (Waltham, Massachusetts). The flg22 peptide from *P. aeruginosa* was used at 0.1 μM as a positive control for the ROS assay.

For the Fla_*Lso*_ assay, *A. tumefaciens* GV3101 containing vector pBin or pBin:*fla*_*Lso*_ with an OD_600_ at 0.5 was used to infiltrate leaves of *N. benthamiana,* tomato and potato as described above. Leaf discs from infiltrated zones were taken on 2, 3, 4, and 5 dpi. ROS assay was performed as described above.

The ion-leakage assay was performed as described [42]. Briefly six leaf discs (5 mm in diameter) were collected at 2, 3, 4, 5 and 8 d with a sharp cork borer after infiltration with 10 mM MgCl2, *A. tumefaciens* containing pBin:fla_*Lso*_ or pBin vector alone as a control, and then washed with 10 mL distilled water for 30 min. Then they were transferred to fresh distilled water. Conductance was measured with an OAKTON electrical conductivity meter (Singapore).

### Reverse transcription quantitative PCR (RT-qPCR)

DNase-treated RNA (~2 μg) was used to synthesize first-strand cDNA with 0.5 μg of oligo (dT) primer and 1 μL of SuperScript® III reverse transcriptase in a 20 μL reaction (Invitrogen). A negative control without the reverse transcriptase was performed to verify the absence of genomic DNA contamination. RT-qPCR was performed with SYBR in triplicate using an Eppendorf Mastercycler® Realplex thermal cycler. The 15 μL amplification reactions contained the following: 7.5 μL of SYBR® Green PCR Master Mix system (PERFECTA SYBR FASTMX LRX, VWR), 250 nM of each forward and reverse primer, and 2.0 μL of diluted cDNA template. The following protocol was used: 95°C for 5 min, 40 cycles of 30 s for denaturation at 95°C and 30 s for extension at 60°C. Primers for *NbFLS2* were designed as 5′-TCAAATGGTGGATGACTGGA-3′ and 5′-ATGATATGCTGCTCCCATCC-3′. The *N. benthamiana* elongation factor 1 alpha (*NbEF1*α) was amplified and used to normalize the values as an internal control with primers (5′-GACCACTGAAGTTGGATCTGTTG-3′; 5′-TAGCACCAGTTGGGTCCTTCTT-3′). All other primers were used as previously reported (*NbWIPK*, *NbSIPK*, *NbRbohB*, *NbACRE31*, *NbACRE32* and *NbCYP71D20* as in publication [14]; *NbPlastocyanin* as in [11]; *NbBAK1* as in [18] and *NbSerk3* as in [26]). For evaluation of transient expression of the *fla*_*Lso*_, tissue collection, RNA isolation and cDNA amplification were carried out as described above. RT-PCR and RT-qPCR were performed to detect the *fla*_*Lso*_ expression levels at various times after infiltration. The primers for *fla*_*Lso*_ were designed as 5′-TTGCGTGTTGCTGATTCTTC-3′ and 5′-TCTGCCTGAACAGAATGTGC-3′. The expression level was normalized against internal control *NbEF1*α. Meanwhile, the 2^_△△CT^ method was used to calculate the changes in relative copy number of the target genes under treated conditions, 2^_△△CT^ method was taken [43] where CT is the point at which the fluorescence signal crosses the threshold and △CT = CT (target gene) - CT (internal control) and △△CT = (CT, Target – CT, internal control) Time x - (CT, Target – CT, internal control) Time x.

## Competing interests

The authors declare that they have no competing interests.

## Authors’ contributions

GH and YPD conceived and designed the experiments. GH and MP performed the experiments. GH, DF and MP analyzed the data. GH, MP and LH contributed reagents/materials/analysis tools. GH, ES and YPD wrote the manuscript. All authors read and approved the final manuscript.

## Additional file

## Supplementary Material

Additional file 1: Figure S1. Electrolyte leakage from leaf discs of *N. benthamiana* leaves inoculated with 10 mM MgCl2, *Agrobacterium tumefaciens* strain GV3101 containing the vector control pBin and the pBin:*fla*_*Lso*_ constructs, respectively. * marked as significant change by student *t*-test. Click here for file
