# Supplementary figures and images for: Induction of innate immune responses by flagellin from the intracellular bacterium, ‘Candidatus Liberibacter solanacearum’
Source: BMC Plant Biol. 2014 Aug 5;14:211. doi: 10.1186/s12870-014-0211-9 (PMC4422280; doi:10.1186/s12870-014-0211-9)

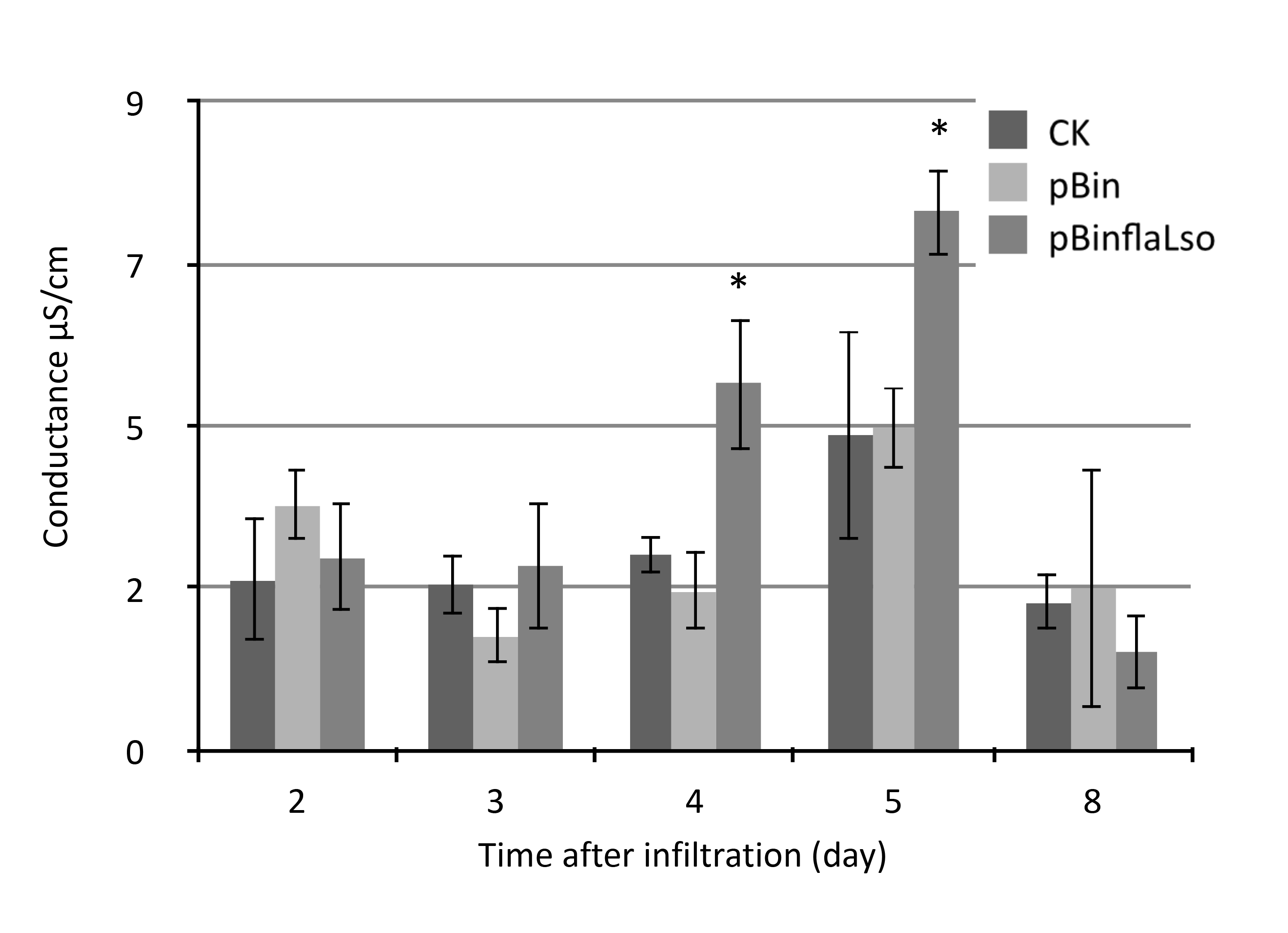

Supplement: Additional file 1: Figure S1. — Electrolyte leakage from leaf discs of N. benthamiana leaves inoculated with 10 mM MgCl2, Agrobacterium tumefaciens strain GV3101 containing the vector control pBin and the pBin:flaLso constructs, respectively. * marked as significant change by student t-test. [file s12870-014-0211-9-S1.tiff]
